# DrugHybrid_BS: Using Hybrid Feature Combined With Bagging-SVM to Predict Potentially Druggable Proteins

**DOI:** 10.3389/fphar.2021.771808

**Published:** 2021-11-30

**Authors:** Yuxin Gong, Bo Liao, Peng Wang, Quan Zou

**Affiliations:** ^1^ School of Mathematics and Statistics, Hainan Normal University, Haikou, China; ^2^ Key Laboratory of Computational Science and Application of Hainan Province, Haikou, China; ^3^ Key Laboratory of Data Science and Smart Education, Hainan Normal University, Ministry of Education, Haikou, China; ^4^ Yangtze Delta Region Institute (Quzhou), University of Electronic Science and Technology of China, Quzhou, China

**Keywords:** monoDiKGap, CC, GAAC, bagging, support vector machine

## Abstract

Drug targets are biological macromolecules or biomolecule structures capable of specifically binding a therapeutic effect with a particular drug or regulating physiological functions. Due to the important value and role of drug targets in recent years, the prediction of potential drug targets has become a research hotspot. The key to the research and development of modern new drugs is first to identify potential drug targets. In this paper, a new predictor, DrugHybrid_BS, is developed based on hybrid features and Bagging-SVM to identify potentially druggable proteins. This method combines the three features of monoDiKGap (k = 2), cross-covariance, and grouped amino acid composition. It removes redundant features and analyses key features through MRMD and MRMD2.0. The cross-validation results show that 96.9944% of the potentially druggable proteins can be accurately identified, and the accuracy of the independent test set has reached 96.5665%. This all means that DrugHybrid_BS has the potential to become a useful predictive tool for druggable proteins. In addition, the hybrid key features can identify 80.0343% of the potentially druggable proteins combined with Bagging-SVM, which indicates the significance of this part of the features for research.

## 1 Introduction

Drug targets refer to the binding sites of drugs in the body. To date, there are approximately 130 protein families as therapeutic drug targets, which usually include enzymes (Liu et al., 2019a; Meng et al., 2020; Xu et al., 2021a; Wang et al., 2021), G protein-coupled receptors (Ru et al., 2020), ion channels and transporters (Han et al., 2019), nuclear hormone receptors, etc (Li and Lai, 2007). These drug targets are of great significance for disease treatment and drug research and development (Ding et al., 2019a; Ding et al., 2019b; Shi et al., 2019; Ding et al., 2020a; Wang et al., 2020a; Ding et al., 2020b; Shang et al., 2021; Zhuang et al., 2021). However, the discovery and development of modern drugs is usually a time-consuming and laborious process. It is estimated that it takes an average of 10–15 years to bring a drug to the market, which costs approximately US $2,558 million (Zhong et al., 2018). Therefore, predicting whether a protein can potentially be used as a drug target has significant value in disease treatment and reducing the time and cost of drug development, which greatly accelerates the drug development process for the protein (Wang et al., 2020b; Yu et al., 2021).

The discovery of drug targets has attracted extensive attention in both academia and the pharmaceutical industry. The commonly used methods for drug target prediction can be roughly divided into three types. The first type is to analyse known drug targets at the genome level based on sequence homology and to find potential drug targets from protein families (Hopkins and Groom, 2002; Russ and Lampel, 2005; Munir et al., 2019; Ao et al., 2021). Not all members of the same protein family can be used as therapeutic drug targets. The second type predicts whether the new target is druggable based on several chemical properties, molecular drug similarity, and target properties (Gayvert et al., 2016). This method is usually limited by experimental cost. The third type is discovering drug targets based on protein structure, which predicts the protein’s drug properties by searching for the binding site and binding affinity of the target protein (Salmaso and Moro, 2018). However, this method has limitations because the three-dimensional structure of most proteins is not easy to obtain.

With the advent of the genome era, revolutionary changes have taken place in the field of drug research and development. Many computing methods were used for effective drug target prediction. To better find potential drug targets and provide new options for drug redirection, Cheng et al. (Cheng et al., 2021) established the GraphMS model. They fused heterogeneous graph information using mutual information in the heterogeneous graph to obtain effective node information and substructure information. The experimental results show that the area under the receiver operating characteristic curve (AUROC) was 0.959, and the area under the precision-recall curve (AUPR) was 0.847. Dezső et al. (Dezső and Ceccarelli, 2020) developed a machine learning model for tumour drug targets. A variety of protein features, including features from sequences, features that characterize protein functions, and network features from protein-protein interaction networks, were included in the model. It has achieved high accuracy on the drug target of independent clinical trial drug targets, with an area under the curve of 0.89. In order to establish a high-quality environment-specific metabolic model that can be used for drug target prediction, Pacheco et al. (Pacheco et al., 2019) developed a metabolic model FASTCORMICS RNA-seq workflow (rFASTCORMICS) based on RNA-seq data. The genes and response characteristics of 13 different types of cancer were extracted. At the same time, 17 new colon cancer candidate drugs were predicted, of which 3 drugs were verified *in vitro* in colon cancer cell lines. Ji et al. (Ji et al., 2019) proposed a DTINet method based on network propagation, starting from the diffusion component analysis of potential drug targets and disease networks. The DTINet performed well under the receiver operating characteristic curve (AUROC = 0.86 ± 0.008). To achieve the rapid identification of novel targets, Li et al. (Li and Lai, 2007) constructed a simple model extraction characteristics from known drug target protein sequences. Using this model, drug targets and nondrug targets can be distinguished with 84% accuracy. Jamali et al. (Jamali et al., 2016) based on the protein features derived from 443 sequences, the accuracy of predicting drug targets through neural network models reached 89.98%.

This paper selected three feature extraction methods: monoDiKGap (k = 2), cross covariance (CC) and grouped amino acid composition (GAAC) (Zuo et al., 2017). The three individual features were mixed in different combinations through the hybrid feature method. The MRMD was used to remove redundant hybrid features, and the integrated method bagging was used to improve the classification performance of potentially druggable proteins. We performed the importance analysis on the best feature combination and selected the key features that distinguish potentially druggable proteins. The results show that the hybrid features of the three feature extraction methods can predict the potentially druggable proteins well by the integrated method bagging, and can correctly predict 96.9944% of the druggable target proteins. This model was conducive to better promotion of drug development. Furthermore, the potential drug targets screened out can provide references for new drug targets.

## 2 Materials and Methods

**FIGURE 1:**
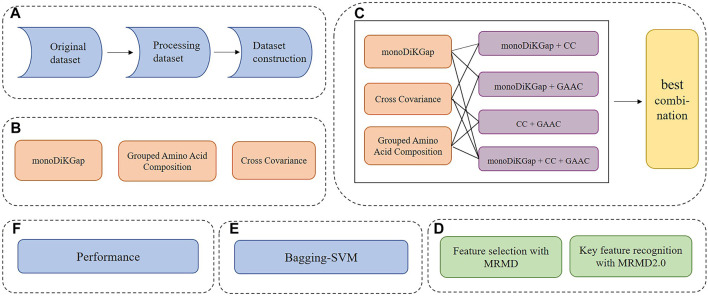
Flow chart of DrugHybrid_BS model **(A)** Process the referenced dataset **(B)** Three single feature representation methods were used to extract features **(C)** Combine three single feature representation methods and select the best hybrid feature **(D)** Use MRMD to remove redundant features and MRMD2.0 to obtain key features **(E)** Feature subsets were used to predict potentially druggable proteins through the optimized Bagging-SVM model **(F)** Evaluate model prediction effects based on performance indicators.

This paper mainly studied the following parts, and the step flow chart was shown in Figure 1:

1. Establishment of dataset.
2. Use three single feature extraction methods, monoDiKGap, Cross Covariance, and Grouped Amino Acid Composition, to represent the features of dataset.
3. Combine three single feature methods to obtain hybrid features.
4. The MRMD was used to remove redundant features, and the MRMD2.0 obtained key features.
5. The feature subset predicted the potentially druggable proteins through the optimized Bagging-SVM model.

This research was carried out under the software python 3.7.4. By comparing the new method DrugHybrid_BS with other machine learning models, the study found that the classification effect of DrugHybrid_BS was better, which was helpful for the prediction of potentially druggable proteins.

### 2.1 Dataset Construction

This paper cited the dataset proposed by Lin et al. (Lin et al., 2019), in which the drug target dataset was downloaded from the DrugBank (Wishart et al., 2006) database. In the original dataset, 1,224 druggable protein sequences were selected as the positive sample set, and 1,319 non-druggable proteins were selected as the negative sample set. We further processed the dataset by removing the protein sequences containing non-standard amino acid characters “B", “J", “O", “U", “X" and “Z". For the remaining sequences, the CD-Hit program (Fu et al., 2012) was used to set a critical value of 60% sequence identity to delete highly similar sequences to avoid overfitting caused by homologous deviation and noise in training (Zou et al., 2020).

**FIGURE 2:**
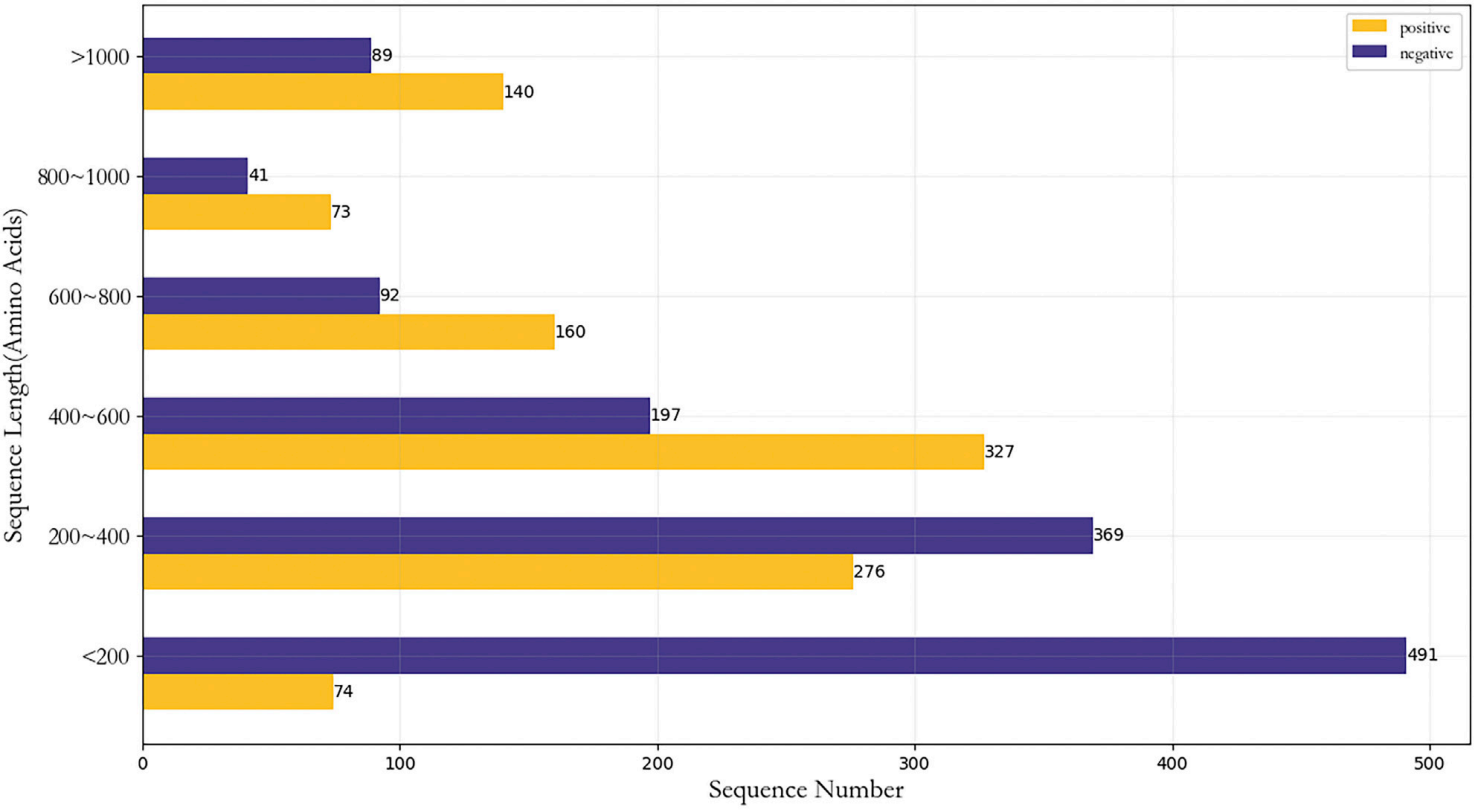
Sample distribution of dataset.

The processed dataset was represented by D, which is the combination of $D^{+}$ and $D^{-}$ :

$$D=D^{+}\cup D^{-}$$ (1)

where $D^{+}$ represents potentially druggable protein samples and $D^{-}$ represents non-druggable protein samples. The positive sample set contained 1,050 protein sequences, and the negative sample set concluded contained 1,279 protein sequences. Figure 2 showed the sample distribution of the dataset.

### 2.2 Feature Representation

#### 2.2.1 monoDiKGap

The monoDiKGap feature is a variant of the kmer feature extraction method in the PyFeat package. Kmer, as our common feature extraction method, is also called k-tuples (Liu et al., 2019b; Lv et al., 2020; Niu et al., 2021a). MonoDiKGap refers to the combination of subsequences with KGap used to describe the sequence. While monoDiKGap generates all feature sets, it can also use the AdaBoost (Zhu et al., 2006) classification model to reduce redundant features to generate the optimal feature set. The generated optimal feature set will not only reduce the feature dimension but also ensure a good prediction. In this study, we set KGap to 2. At this time, the monoDiKGap feature can be expressed as:

$$V_{\mathrm{KGap}}={\left[f_{1}^{k_{1}},f_{2}^{k_{1}},...,f_{8000}^{k_{1}},f_{1}^{k_{2}},f_{2}^{k_{2}},...,f_{8000}^{k_{2}}\right]}^{T}$$ (2)

where $f_{i}^{k_{1}}\left(i=1,2,...,8000\right)$ represents the frequency of the *i*th feature calculated when the feature was shaped like X_XX , and the generated feature at this time was like "A_AA" . $f_{i}^{k_{2}}\left(i=1,2,...,8000\right)$ represents the frequency of the *i*th feature calculated when the feature was shaped like $X_{——}XX$ , the generated feature was like $"A_{——}AA"$ , and X represents twenty natural amino acids. Therefore, the total feature set generated by this feature extraction method has a total of 16,000 features, which AdaBoost automatically optimizes to generate 466 feature subsets with more discriminative capabilities.

#### 2.2.2 Cross Covariance (CC)

CC is the correlation between two different attributes separated by lag (Guo et al., 2008). For this study, the CC variable described the average interaction between two fragments with different physical and chemical properties separated by lag fragments. Suppose that the protein sequence P has L residues, $P=R_{1}R_{2}R_{3}...R_{L}$ . where $R_{i}\in \left\{A,C,D,E,F,G,H,I,K,L,M,N,P,Q,R,S,T,V,W,Y\right\}$ represents the amino acid at position (i=1,2,...,L) in the sequence. Then, for each protein sequence, there is a physical and chemical information matrix of the following L×3 size, which can be expressed as:

$$X=\left[\begin{array}{ccc} x_{11} & x_{12} & x_{13}\\ \begin{array}{l} x_{21}\\ ... \end{array} & \begin{array}{l} x_{22}\\ ... \end{array} & \begin{array}{l} x_{23}\\ ... \end{array}\\ x_{L1} & x_{L2} & x_{L3} \end{array}\right]$$ (3)

where $x_{i1,}x_{i2},x_{i3}\left(i=1,2,...,L\right)$ stands for the hydrophobicity values, hydrophilicity values and side chain mass of amino acid $R_{i}$ , respectively.

CC converts protein sequences of different lengths into feature vectors of the same length. The calculation formula of the CC feature representation method is as follows:

$$\mathrm{CC}\left(k,j,lag\right)=\sum_{i=1}^{L-lag}\left(x_{i,k}-\bar{x_{k}}\right)\left(x_{i+lag,j}-\bar{x_{j}}\right)$$ (4)

lag=1,2,...,lg , here lg=2 was the default. Because CC was an asymmetric vector, under this physical and chemical characteristic condition, the feature dimension of the CC vector was twelve.

#### 2.2.3 Grouped Amino Acid Composition (GAAC)

In the GAAC code, twenty amino acid types are divided into five categories based on their physical and chemical properties (Lee et al., 2011; Zheng et al., 2019; Zheng et al., 2021). These five categories include the aliphatic group $\left(g_{1}:\mathrm{GAVLMI}\right)$ , aromatic group $\left(g_{2}:\mathrm{FYW}\right)$ , positive charge group (g3:KRH) , negative charged group $\left(g_{4}:\mathrm{DE}\right)$ , and uncharged group $\left(g_{5}:\mathrm{STCPNQ}\right)$ .

The GAAC descriptor refers to the frequency of each amino acid group, which is calculated as follows:

$$f\left(g\right)=\frac{N\left(g\right)}{N},g\in \left\{g_{1},g_{2},g_{3},g_{4},g_{5}\right\}$$ (5)

$$N\left(g_{t}\right)=\sum N\left(t\right),t\in g$$ (6)

where N(g) is the number of amino acids in group g , N(t) is the number of amino acid types t , and N is the length of the protein sequence.

As an example, for the sequence "EAHGAFLMDKPSMFNERV" , the amount of occurrences of character “E" was 2, the amount of occurrences of character “A" was 2, the amount of occurrences of character “H" was 1, the amount of occurrences of character “G" was 1, etc. The length of the sequence was 18, $N\left(g_{1}\right)=7,N\left(g_{2}\right)=2,N\left(g_{3}\right)=3,N\left(g_{4}\right)=3,N\left(g_{5}\right)=3$ . Therefore, the GAAC feature of this sequence was expressed as $\left(\frac{7}{18},\frac{1}{9},\frac{3}{18},\frac{3}{18},\frac{3}{18}\right)$ .

### 2.3 Machine Learning Algorithm

In this study, predicting druggable proteins was a typical binary classification problem. To better explore prediction models and analysis features, we mainly used four machine learning algorithms for prediction tasks, namely, support vector machine, K-nearest neighbour, bagging integrated learning, and random forest.

#### 2.3.1 K-Nearest Neighbour (KNN)

The k-nearest neighbour algorithm is a classic machine learning algorithm (Liao and Vemuri, 2002; Samanthula et al., 2014). The principle of the k-nearest neighbour algorithm is straightforward: a sample in the feature space will always find the k data closest to it, that is, the nearest sample in the feature space. If most of the k data belong to a specific category, the sample also belongs to this category. In this study, the default parameters of the prediction model were selected, and the value of k was 3.

#### 2.3.2 Support Vector Machine (SVM)

Although the support vector machine has only a short development history of more than 20 years. It shows strong energy in classification problems (Ding et al., 2017; Wei et al., 2018a; Wang et al., 2019; Wang et al., 2020c; Huo et al., 2020). It has become the mainstream technology of machine learning from the end of the 20th century to the beginning of the 21st century, applied to many fields (Jiang et al., 2013; Xu et al., 2018; Zhang et al., 2018; Wei et al., 2019a; Liu et al., 2021). The support vector machine uses the maximum classification interval to determine the optimal partitioning hyperplane to obtain good generalization. For the binary classification problem in this study, when we obtain a feature dataset containing category information:

$$S=\left\{\left(x_{1,}y_{1}\right),\left(x_{2},y_{2}\right),...,\left(x_{n},y_{n}\right)\right\},y_{i}\in \left\{1,-1\right\}$$ (7)

where n was the number of samples, the feature dimension of each sample was d, and the samples were divided into positive categories ( $y_{i}=1$ represents druggable protein) and negative categories ( $y_{i}=-1$ represents non-druggable protein). Our goal was to find the optimal hyperplane to maximize the sample interval between the positive class and the negative class.

We used $\omega^{T}x+b=0$ to represent the partitioning hyperplane and used the geometric margin to find the optimal partitioning hyperplane. The geometric interval was numerically equal to the distance from the sample point to the partition hyperplane. The distance from the positive sample point $\left(x_{i},y_{i}=1\right)$ to the partition hyperplane was $\frac{\omega^{T}x_{i}+b}{{\left\|\omega \right\|}_{2}}$ , and the distance from the negative sample $\left(x_{i},y_{i}=-1\right)$ to the partition hyperplane was $\frac{-\left(\omega^{T}x_{i}+b\right)}{{\left\|\omega \right\|}_{2}}$ , where ω was the normal vector of the partition hyperplane and b was the intercept. Therefore, the distance from any sample $\left(x_{i},y_{i}\right)$ to the partition hyperplane can be uniformly expressed as $\frac{y_{i}\left(\omega^{T}x_{i}+b\right)}{{\left\|\omega \right\|}_{2}}$ . To solve the optimization problem of linear separable support vector machines. John C. Platt proposed the sequential minimal optimization algorithm (Platt, 1998) in 1998. The algorithm decomposed the large convex quadratic programming (QP) problem to be solved in the training process of support vector machines into a series of minimum possible QP problems, avoided time-consuming internal iterative optimization, and improves computational efficiency.

In addition, the kernel function is a unique feature of the support vector model. For the same dataset, different kernel function choices will have different prediction effects. Appropriate kernel functions can improve prediction performance. The commonly used functions include the linear, Gaussian, and polynomial kernel functions. In this study, a linear kernel function was selected as the kernel of the support vector machine by comparing different kernel functions.

#### 2.3.3 Bagging

Bagging is one of the common ensemble learning models (Dudoit and Fridlyand, 2003; Jin et al., 2019; Jin et al., 2021; Wu and Yu, 2021). The ensemble learning model uses a series of weak learners (also called basic models) for learning and integrates the results of each weak learner to obtain a better learning effect than individual learners.

The bagging algorithm uses the simplest combination strategy to obtain the integration model. For the classification problem, the majority voting method is adopted. Each weak learner has one vote, and the final prediction result is generated according to the votes of all weak learners. The process of the bagging method is as follows: suppose we have a training set containing N samples and randomly put back the data to form a new training set. Because there is a way to put back sampling, a sample may be selected multiple times, or a sample may not be selected once. Hence, the size of the sampled data samples is the same as that of the original training data samples, but they contain different data. In this way, after T groups of data are extracted, T weak learners trained by different training sets can be obtained at the end of training. According to the prediction results of T weak learners, the most voting method is adopted to obtain a more accurate and reasonable prediction model.

#### 2.3.4 Random Forest(RF)

Random forest is a representative bagging algorithm based on decision trees. Because random forest has good performance in regression and classification prediction, it has attracted great attention. It has been widely used in many practical problems, such as genome data analysis and disease risk prediction. When making classification prediction, each decision tree will make classification judgment on the data according to the characteristics of the data. Through the majority voting method, the category with the most votes is the prediction result of the random forest.

### 2.4 Feature Selection

In the feature extraction section, we introduced three feature representation methods. The optimal feature subset of the dataset sample generated by the monoDiKGap method had 466 features. The CC feature representation method generated 12 features, and the GAAC feature method generated five features. Different feature extraction methods were combined to obtain hybrid features. However, the hybrid of features may lead to feature redundancy and affect the predictive effect of potentially druggable proteins. Therefore, we used MRMD and MRMD2.0 to select features and used fewer features to distinguish between potentially druggable and non-druggable proteins better.

In this study, the MRMD (Quan et al., 2016) was used to remove redundant features in hybrid features. The MRMD will leave the optimal feature subset after automatic feature selection. The main principle of this method is to use the Euclidean distance, cosine distance, and the Tanimoto coefficient to calculate the redundancy between features and use the Pearson correlation coefficient to calculate the correlation between dataset features and class labels to generate feature subsets with low redundancy and strong correlation automatically. When we analyse the hybrid feature subset that can accurately predict potentially druggable proteins, we also need to analyse the importance of different features. MRMD2.0 (He et al., 2021) combined seven algorithms, such as ANOVA, MIC, LASSO, mRMR, and chi-square test, through the PageRank strategy algorithm to rank different algorithm lists to form a directed graph, and each feature obtained a score. According to the ranking information, we analyse the importance of features and obtain key features that influence the prediction of potentially druggable proteins.

### 2.5 Performance Evaluation

To intuitively measure the quality of the model, we evaluated the predictive effect of the model. This study used common evaluation indicators, including TP rate (TPR), FP rate (FPR), precision (Su et al., 2018), F-score (Sokolova et al., 2006), and accuracy (ACC) (Wei et al., 2017a; Wei et al., 2017b; Wei et al., 2018b; Wei et al., 2019b; Huang et al., 2020; Liang et al., 2020; Zhang et al., 2020; Xu et al., 2021b; Zhu et al., 2021). The calculation method of each measurement index was as follows:

$$\left\{ \begin{array}{l} \mathrm{TPR}=\frac{\mathrm{TP}}{\mathrm{TP}+\mathrm{FN}}\\ \mathrm{FPR}=\frac{\mathrm{FP}}{\mathrm{FP}+\mathrm{TN}}\\ \mathrm{precision}=\frac{\mathrm{TP}}{\mathrm{TP}+\mathrm{FP}}\\ \mathrm{recall}=\frac{\mathrm{TP}}{\mathrm{TP}+\mathrm{FN}}\\ F_{\mathrm{score}}=\frac{2*\mathrm{precision}*\mathrm{recall}}{\mathrm{precision}+\mathrm{recall}}\\ \mathrm{ACC}=\frac{\mathrm{TP}+\mathrm{TN}}{\mathrm{TP}+\mathrm{FP}+\mathrm{TN}+\mathrm{FN}} \end{array}\right.$$ (9)

Here, TP represents the classification number of correct positive samples, and TN represents the classification number of correct negative samples. FP represents the classification number of false positive samples. FN represents the classification number of false negative samples. In addition, this study also used 5-fold cross-validation to predict and evaluate the model.

## 3 Results and Discussion

### 3.1 Performance of Single Feature Extraction Methods

**FIGURE 3:**
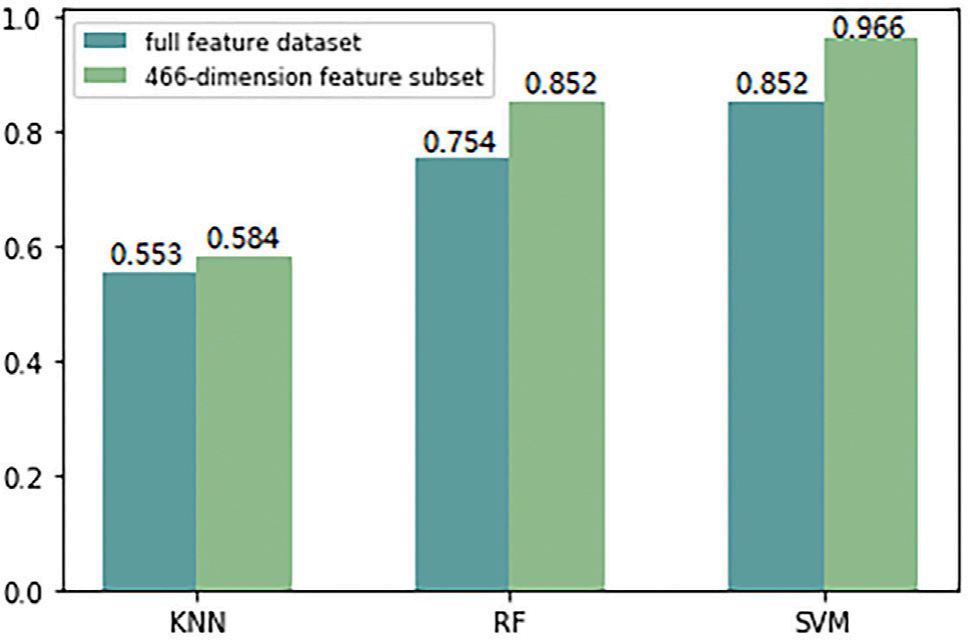
Comparison the ACC values of the full feature set and 466-dimensional feature subset extracted by monoDiKGap(k = 2) under different classifiers.

Because the monoDiKGap feature extraction method gradually increases with the value of KGap, the number of corresponding generated feature vectors increases exponentially. In this study, the total feature set generated by monoDiKGap(k = 2) has 16,000 features, but in fact many small fragments appear very rarely, and some even appear 0 or 1 times. At this time, a large number of feature vectors composed of 0 or one also have no meaning already. In order to avoid high-dimensional feature vectors introducing dimensional disasters for subsequent machine learning algorithms, resulting in a significant decline in predictive classification performance. Therefore, this study used AdaBoost to automatically generate a more discriminative 466-dimensional feature subset, and compared the ACC values of the full feature set and the feature subset of monoDiKGap(k = 2) under different classifiers, as shown in Figure 3.

In this paper, three single feature extraction methods, monoDiKGap(k = 2), CC and GAAC, were used to represent the features of the dataset. Three single feature representation methods extracted 466-dimensional, 12-dimensional, and 5-dimensional features. The prediction performance of each extraction method under SVM, KNN, and RF was shown in Table 1. The data in Table 1 showed that the accuracy of the monoDiKGap(k = 2) feature representation method in predicting potentially druggable proteins through the SVM classification algorithm was higher than that of KNN and RF. The model can accurately predict 96.608% of the potentially druggable proteins. At this time, the TPR value reached 0.965, the FPR value reached 0.033, the F-score reached 0.962, and the ROC curve area was 0.966. The GAAC feature extraction method had an accuracy of 77.2864% in predicting potentially druggable proteins under SVM, which was 1.20 and 2.40% higher than that of the RF and KNN classification models, respectively. The accuracy of the CC feature extraction method to predict proteins through the SVM feature representation method was only 1.07% lower than that of the KNN algorithm. Therefore, considering the performance evaluation of the three feature representation methods under different classifiers, the SVM classification algorithm was more suitable for accurately predicting potentially druggable proteins.

**TABLE 1:** Compare the results of different feature methods under different classifiers.

Method	Classifier	ACC(%)	TPR	FPR	Precision	F-score	auROC
monoDiKGap (k = 2)	SVM	96.608	0.965	0.033	0.960	0.962	0.966
	KNN	58.437	0.083	0.004	0.946	0.152	0.628
	RF	85.272	0.788	0.094	0.873	0.828	0.928
CC	SVM	57.364	0.243	0.155	0.563	0.339	0.544
	KNN	58.437	0.625	0.449	0.533	0.575	0.599
	RF	63.718	0.569	0.306	0.604	0.586	0.679
GAAC	SVM	77.286	0.768	0.223	0.739	0.753	0.772
	KNN	74.882	0.745	0.248	0.712	0.728	0.807
	RF	76.084	0.729	0.213	0.738	0.733	0.850

### 3.2 Performance of Hybrid Feature Representation Methods

To explore the prediction performance of hybrid features, we combined the above three feature representation methods and obtained new feature vectors of different combinations. After the combination of three single feature extraction methods, four new feature vectors were obtained: monoDiKGap + CC, monoDiKGap + GAAC, CC + GAAC and monoDiKGap + CC + GAAC. Table 2 showed the evaluation performance of different combinations of hybrid features using the SVM classification algorithm. Table 2 indicated that compared with the single feature representation method, the hybrid feature showed higher performance. The accuracy of the combination of monoDiKGap and other feature representation methods was more than 96%. In addition, the prediction performance of the CC + GAAC feature combination was also higher than that of the single feature representation method. Importantly, we found that the combination of monoDiKGap, CC, and GAAC features showed the best prediction performance, and the hybrid feature could accurately predict 96.6509% of potentially druggable proteins.

**TABLE 2:** Performance comparison of different feature combinations under SVM classifiers.

Method	ACC(%)	TPR	FPR	Precision	F-score	auROC
monoDiKGap + CC	96.651	0.967	0.034	0.959	0.963	0.967
monoDiKGap + GAAC	96.350	0.958	0.032	0.961	0.959	0.963
CC + GAAC	78.360	0.770	0.206	0.755	0.801	0.782
monoDiKGap + CC + GAAC	96.651	0.961	0.029	0.965	0.963	0.966

### 3.3 Kernel and Parameters of Support Vector Machine

**FIGURE 4:**
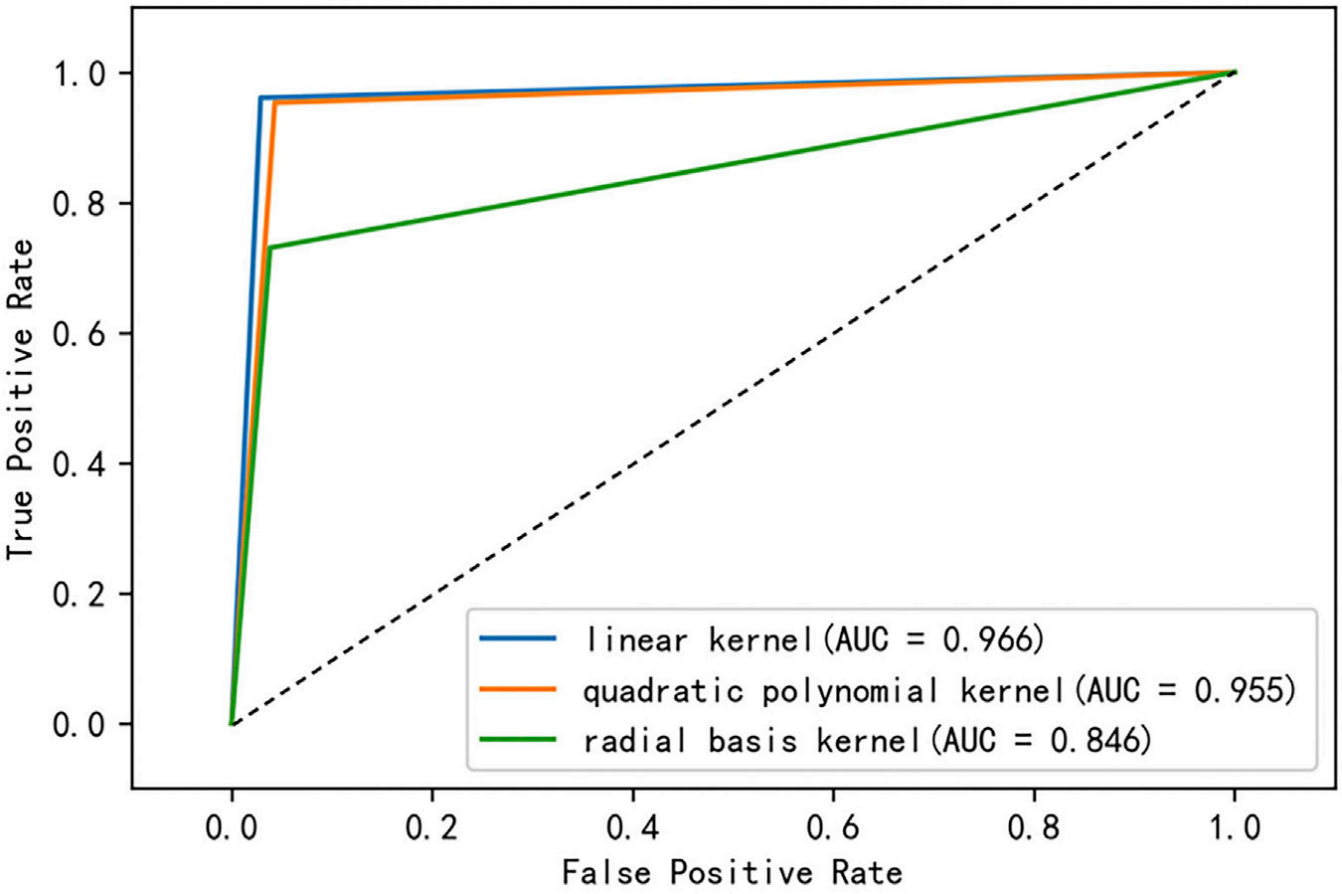
ROC curves of support vector machines in different kernel functions.

The kernel function is an important feature of support vector machines. The kernel function choice of the support vector machine affects the prediction performance of the model. For the monoDiKGap, CC, and GAAC hybrid features to represent the dataset features, we used different kernel functions and 5-fold cross-validation to select the appropriate kernel function. We compared the performance of the linear kernel function, quadratic polynomial kernel function, and radial basis kernel function. The ROC curves of different kernel functions were shown in Figure 4. The ROC values were 0.966, 0.955, and 0.846. The evaluation indicators of the three kernel functions were shown in Table 3. We can see that the prediction effect of the hybrid feature using the linear kernel function was better than the quadratic kernel function and the radial basis function. At this time, the three kernel functions predicted 96.6509, 95.5346, and 85.745% of the potentially druggable proteins, respectively. Therefore, this paper chose a linear kernel function as the kernel of the support vector machine.

**TABLE 3:** Performance comparison of hybrid features under different kernel functions.

Kernel function	ACC(%)	TPR	FPR	Precision	F-score	auROC
liner kernel	96.651	0.961	0.029	0.965	0.963	0.966
polynomial kernel	95.535	0.953	0.043	0.948	0.951	0.955
RBF	85.745	0.730	0.038	0.940	0.822	0.846

For the linear kernel of the support vector machine, the penalty parameter C is an important parameter. The larger the value of C is, the easier it is to overfit, while the smaller the value of C is, the easier it is to underfit. The most commonly used C values are 1, 10, 100, and 1,000. We selected the appropriate C value with the help of grid search. Table 4 showed the prediction performance of different penalty parameters. When the C value was 1, the support vector machine classification algorithm achieved a better prediction effect and shortened the running time.

**TABLE 4:** Performance comparison of hybrid features with different penalty parameter C values under linear kernel.

C Values	ACC(%)	TPR	FPR	Precision	F-score	auROC
1	96.651	0.961	0.029	0.965	0.963	0.966
10	96.651	0.960	0.030	0.965	0.963	0.966
100	96.608	0.960	0.029	0.965	0.962	0.966
1,000	96.608	0.960	0.029	0.965	0.962	0.966

### 3.4 Hybrid Feature Selection

The best hybrid features are 483-dimensional features mixed by the monoDiKGap, CC, and GAAC feature representation methods. These features may contain redundancy and affect the performance. Since the monoDiKGap feature extraction method automatically generated the optimal feature subset, we also need to remove redundant features from the CC and GAAC feature extraction methods. We used MRMD to filter the feature sets extracted by CC and GAAC and generated the optimal feature subset with low redundancy and strong correlation. Finally, we combined the feature subsets to obtain the filtered new hybrid features. These hybrid features not only reduced the feature dimensions but also had more expressiveness (Table 5).

**TABLE 5:** Comparison of classification performance of hybrid features before and after using MRMD feature selection.

Number of feature	ACC(%)	TPR	FPR	Precision	F-score	auROC
483	96.651	0.961	0.029	0.965	0.963	0.966
472	96.694	0.959	0.027	0.967	0.963	0.966

### 3.5 Bagging Algorithm and Comparison With Other Algorithms

The expressive ability of a single support vector machine classification model may be limited so that the bagging ensemble algorithm based on a support vector machine has room for improvement. Compared with a single model, the bagging integration method can enhance the expressive ability of the model and reduce the error. When it is difficult for a single model to correctly distinguish the two types of data, the ensemble algorithm can often improve the model’s prediction performance by constructing multiple independent base models.

**FIGURE 5:**
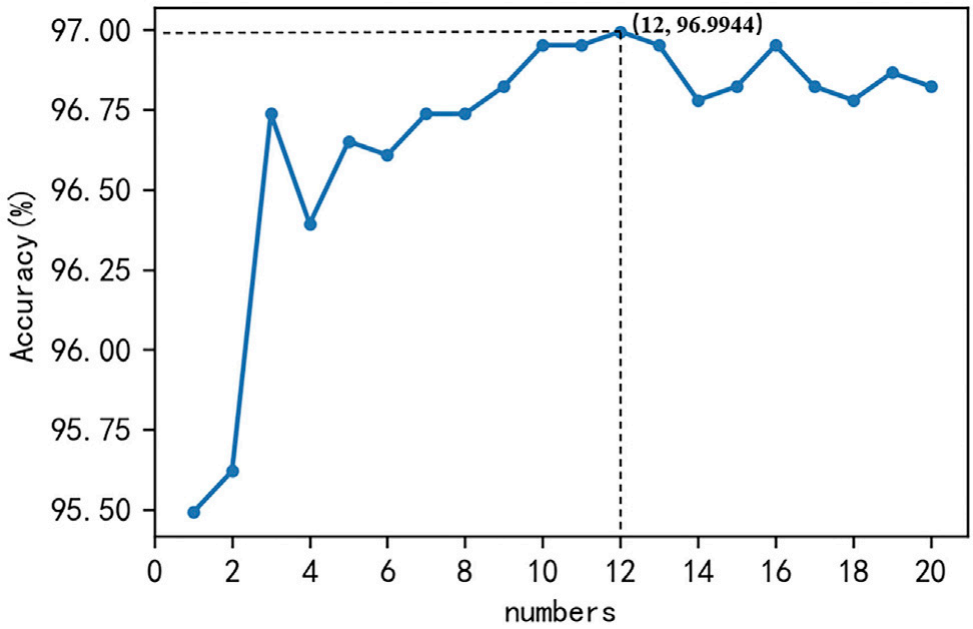
The accuracy of hybrid features in predicting potential druggable proteins under the Bagging-SVM classification algorithm where the number of base models was 1–20.

In this study, a support vector machine with a penalty coefficient of one and a linear kernel function was used as the basic model, and the number of optimal basic models was selected to construct a Bagging-SVM classification algorithm. The hybrid features of monoDiKGap, CC, and GAAC, which removed the cumbersome features, were shown in Figure 5 under the Bagging-SVM classification algorithm where the number of base models was 1–20. The accuracy of combining hybrid features and Bagging-SVM to predict potentially druggable proteins was basically more than 96.73%, and the highest prediction accuracy was 96.9944% when the number of base models was 12.

Based on the hybrid features of monoDiKGap, CC, GAAC, and Bagging-SVM, a new predictive model, DrugHybrid_BS, was constructed. To further explore the prediction model, we evaluated the performance of SVM, RF, and KNN using the same hybrid feature set. Table 6 showed that the DrugHybrid_BS model can better predict potentially druggable proteins. At this time, the TPR value reached 0.970, the F-score reached 0.967, and the AUC value reached 0.992. In addition, Table 6 showed the prediction performance comparison between the DrugHybrid_BS model and the previous model when using the same dataset as Lin et al. (Lin et al., 2019) and Jamali et al. (Jamali et al., 2016). The study found that the accuracy of the original data set using the DrugHybrid_BS model reached 100%, which shows that the original data does have redundancy, and it also reflects the significance of the initial data preprocessing in this article.

**TABLE 6:** Comparison of prediction performance with other algorithms.

Method	ACC(%)	TPR	FPR	Precision	F-score	auROC
DrugHybrid_BS(This paper)	96.994	0.970	0.030	0.963	0.967	0.992
DrugHybrid_KNN	58.652	0.587	0.502	0.729	0.473	0.625
DrugHybrid_SVM	96.694	0.959	0.027	0.967	0.963	0.966
DrugHybrid_RF	87.763	0.834	0.087	0.888	0.860	0.949
DrugHybrid_BS(Original dataset)	100	1.000	0.000	1.000	1.000	1.000
Jamali et al. (Jamali et al., 2016) (Original dataset)	89.78	0.901	0.106	0.901	0.901	0.959
Lin et al. (Lin et al., 2019) (Original dataset)	93.78	0.928	0.056	0.942	0.936	0.978

### 3.6 Independent Test Set

**FIGURE 6:**
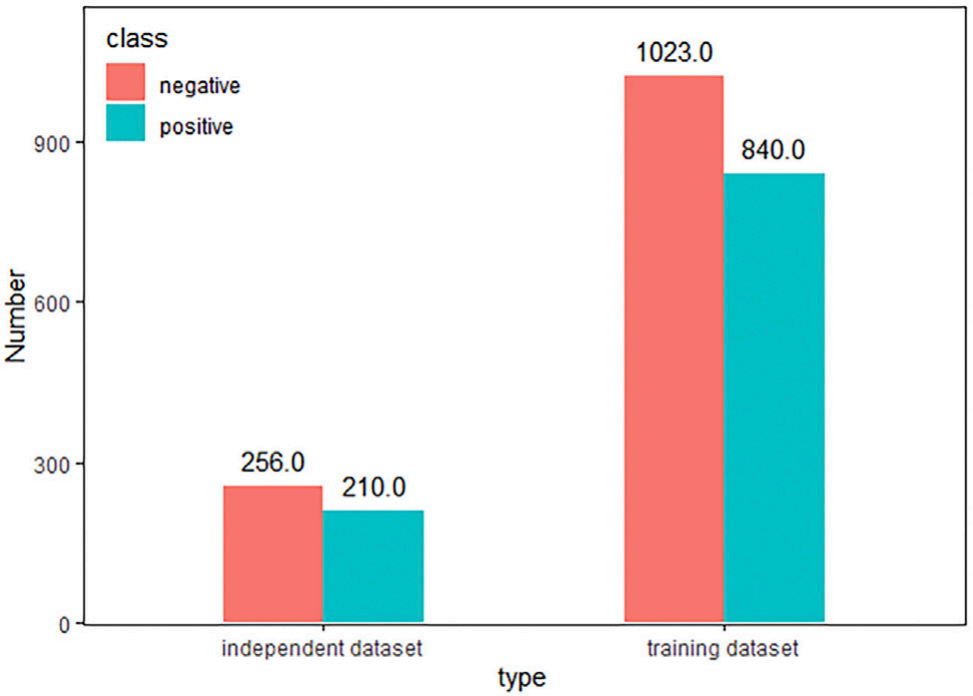
Details of the training set and independent test set.

The accuracy of the classification and prediction model in predicting the training set cannot well reflect the future performance of the prediction model. To effectively judge the performance of a predictive model, we divided 80% of the dataset as the training set and 20% as the test set. The detailed information was shown in Figure 6. The independent test set using the DrugHybrid_BS model can accurately predict 96.5665% of the potentially druggable protein. The TPR value was 0.948, the FPR value was 0.02, the precision value was 0.975, and the AUC value was 0.990.

### 3.7 Feature Importance Analysis

From the DrugHybrid_BS model, we obtained the following: after combining the single feature representation methods, the hybrid features of monoDiKGap, CC and GAAC combined with Bagging-SVM can improve the accuracy of predicting druggable proteins. This part further explored the features that play a key role in the DrugHybrid_BS model, that is, the importance of these features.

**FIGURE 7:**
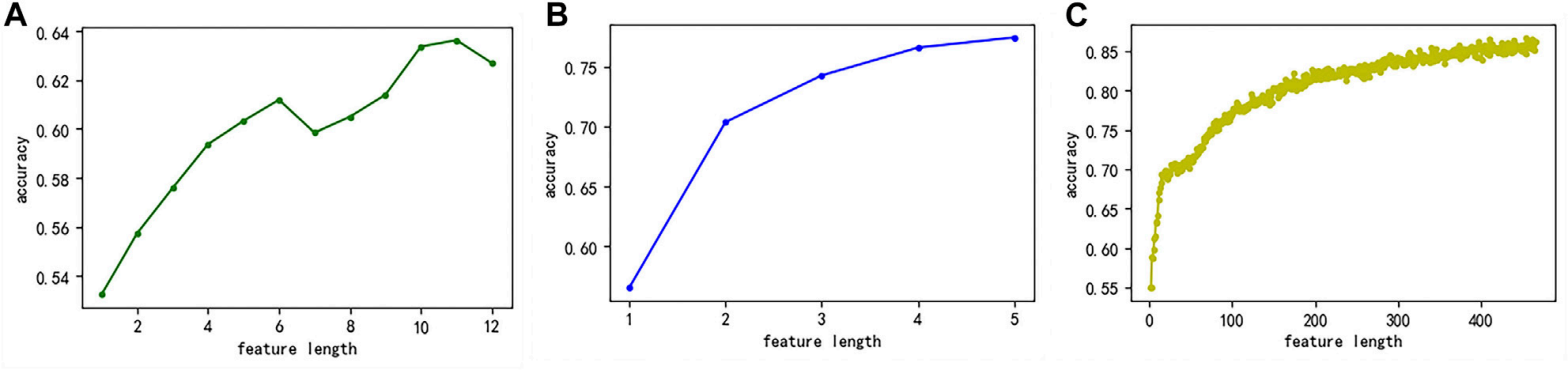
The relationship between the number of features extracted by the three methods and the accuracy of predicting potentially druggable proteins **(A)** CC **(B)** GAAC, and **(C)** monoDiKGap.

First, we used the MRMD2.0 to sort the feature sets extracted by three single feature representation methods and simultaneously obtained the relationship between the number of features and the accuracy of predicting potential druggable proteins (Figures 7A–C). Figure 7A showed that when the number of features of the CC feature extraction method was more than eight, the accuracy rate reached more than 60% and continued to grow. Therefore, we selected the top eight features as the key features of the CC feature representation method. Figure 7B showed the GAAC feature representation method. When the number of features was two, the accuracy rate reached more than 70%, and the accuracy rate continued to increase as the number increased. Therefore, we selected the top two features as the key features of the GAAC feature extraction method. Figure 7C showed the monoDiKGap feature extraction method. When the number of features was twenty-six, the accuracy of predicting potentially druggable proteins was significantly improved, and then the accuracy increased steadily as the number of features increased. Therefore, we chose the top twenty-six features as the key features of the monoDiKGap feature extraction method. Second, we combined the key features of the single feature extraction methods to obtain the hybrid key features. The detailed information was shown in Table 7. Finally, the number of base models suitable for the hybrid key features was selected through the Bagging-SVM classification model.

**TABLE 7:** Key feature details of each feature representation method.

Method	Key features								
GAAC	Aromatic group			Uncharge group					
CC	(mass, hydrophobicity,1)			(mass, hydrophilicity,1)			(hydrophilicity, mass,1)		
	(mass, hydrophobicity,2)			(hydrophobicity, mass,2)			(hydrophobicity, mass,1)		
	(hydrophilicity, mass,2)			(hydrophilicity, hydrophobicity,1)					
monoDiKGap	C_ _NQ	C_ _RT	E_ DT		W_ _PR	E_ _VW		T_ _IL	T_ _PN
	I_RH	Q_ _SA	K_ _IY		L_ _HY	N_TD		T_YK	E_ _DI
	Y_ _LI	R_ _MH	T_ _YY		N_DD	P_RQ		R_ _CT	S_ _GL
	E_VC	P_NY	D_KK		N_PK	F_ _LK		—	—

After research, we obtained that the hybrid key features can accurately predict 80.0343% of the potentially druggable proteins under the bagging algorithm based on the integration of fifteen SVMs. These hybrid key features combined with Bagging-SVM have achieved good prediction results, which fully demonstrated the importance of this part of the feature for the new method DrugHybrid_BS for predicting potentially druggable proteins.

## 4 Conclusion

Research on potentially druggable proteins is of great significance in the field of drug development and disease treatment. However, identifying potentially druggable proteins is the first step in research. This research focused on combining hybrid features and Bagging-SVM to predict potentially druggable proteins. The hybrid features included three feature extraction methods: monoDiKGap, CC, and GAAC, which were based on sequence information, physiochemical properties, and correlation. Through the three single feature representation methods of monoDiKGap, CC, GAAC, and the comparison of combined feature prediction, it was found that the hybrid features of monoDiKGap, CC, and GAAC can accurately predict 96.9944% of the potentially druggable proteins under Bagging-SVM. In addition, the accuracy of the independent test set using the new method DrugHybrid_BS reached 96.5665%. Therefore, the DrugHybrid_BS model used in this study could be a powerful method to study potentially druggable proteins and provide a reference value for other studies. In the future, we will try more deep learning techniques (Zou et al., 2019; Guo et al., 2020; Zeng et al., 2020; Niu et al., 2021b; Zhang et al., 2021) for this problem.

## Data Availability

The original contributions presented in the study are included in the article/Supplementary Material, further inquiries can be directed to the corresponding author.
